# The Effect of the Species and Harvesting Location on Dried Salted Cod Fatty Acid Signatures and Nutritional Quality

**DOI:** 10.3390/foods12030654

**Published:** 2023-02-02

**Authors:** Mário Quaresma, Gonçalo Pereira, Maria Leonor Nunes, Angela Jardim, Carlos Santos, Narcisa Bandarra, Cristina Roseiro

**Affiliations:** 1 CIISA—Centre for Interdisciplinary Research in Animal Health, Faculty of Veterinary Medicine, University of Lisbon, 1300-477 Lisbon, Portugal; 2 AL4AnimalS—Associate Laboratory for Animal and Veterinary Sciences, 1300-477 Lisbon, Portugal; 3 CIIMAR—Interdisciplinary Centre of Marine and Environmental Research, University of Porto, Terminal de Cruzeiros do Porto de Leixões, Av. General Norton de Matos S/N, 4450-208 Matosinhos, Portugal; 4 DGAV—General Directorate of Food and Veterinary, Food and Veterinary Division of Setúbal, 2900-315 Setúbal, Portugal; 5 INIAV, IP—Food Technology and Safety Division, National Institute for Agricultural and Veterinary Research, Quinta do Marquês, Av. da República, 2780-159 Oeiras, Portugal; 6 IPMA, IP—Division of Aquaculture and Upgrading, Portuguese Institute for the Sea and Atmosphere, Av. Alfredo Magalhães Ramalho, 6, 1495-165 Algés, Portugal

**Keywords:** *Gadus morhua*, *Gadus macrocephalus*, dried salted cod, cholesterol, total lipid, fatty acids, canonical discriminant analysis

## Abstract

The Atlantic cod was listed as ‘vulnerable’ by the International Union for Conservation of Nature, a condition that persists today. Fishing pressure on the Atlantic cod could be partially transferred to the Pacific cod, since the two cod species share genetic and phenotypic similarities. The aim of this study is to expand knowledge of the composition of dried salted cod obtained from Atlantic and Pacific cod species, with the Atlantic cod being from two different harvesting locations. The comparison of these cod species revealed the existence of nine significant differences among individual FAs (accountable for 63.2% of total FAs), which was at a similar level to that observed between different harvesting locations for the Atlantic cod (ten significant differences among individual FAs, accountable for 61.6% of total FAs). Canonical discriminant analysis and cross-validation achieved full discrimination of the cod’s origin and 100% accuracy in the cod’s origin classification. The amount of EPA plus DHA in dried salted cod reached its higher value among the Pacific cod (302.3 mg/100 g), while the Atlantic cod averaged 284.1 g/100 g of edible portion. The Pacific cod presented a higher α-tocopherol content than its Atlantic counterpart (8.04 vs. 4.94 µg/g).

## 1. Introduction

The Atlantic cod (*Gadus morhua*) was among the ten most landed species worldwide between 1950 and 2018 [1], and is the third most consumed fish species in the European Union, after tuna and salmon, being associated with a 9% share of total seafood consumption, with a per capita consumption of 2.1 kg [2].

Dried and dried salted cod have been considered value-added products due to their high nutritional value (high bioavailable protein) and specific sensory properties (texture and unique flavour) imparted by the processing technologies [3]. Salting and drying of fresh cod were developed to obtain a shelf-stable product, yet are largely used nowadays to provide sensorial characteristics much valued by the consumer [3].

The consumption of cod products dates back to ancient times. Based on zooarchaeological data obtained on Sanak Island of Alaska, it was estimated that human consumption of Pacific cod (*Gadus macrocephalus*) began 4500 years ago [4]. The use of Atlantic cod (*Gadus morhua*) in the human diet is much more recent, and the first zooarchaeological evidences date back to the 9^th^ century, and were found in Iceland and Norway [5]. Beyond self-provision, the Lofoten and Vesteralen islands in the Norwegian Arctic became the production centre of air-dried fish (Atlantic herring and cod) in the 13th century, supplying populations throughout Northern and Central Europe [6]. The Vikings were, therefore, considered pioneers in the discovery and use of the Atlantic cod, although their preservation method relied exclusively on air-drying, since they had no easy access to salt [5]. The warmer climates in the countries of Southern Europe made the storage of air-dried cod a hazardous situation. Thus, in order to minimize biological risks, the Basque community started to introduce salting before drying [7]. The consumption of dried salted cod has persisted until today, being primarily consumed in the Mediterranean countries (Portugal, Spain and Italy) and in Latin America [8]. During cod salting and ripening periods, several changes occur in the edible portion at both flavour and textural levels, which persist through cooking [9]. The flavour of dried salted cod results from a complex combination of enzymatic and/or chemical reactions, such as lipid oxidation, and Maillard and Strecker degradation reactions [9,10], which impart the formation of numerous volatile compounds with odorous properties [11]. Moreover, salting improves the flavour because it decreases water activity, which can lead to an effective increase in the concentration of flavour components and improve its volatility [12]. Additionally, it increases the water holding capacity (WHC) and consequently its juiciness [9].

Atlantic cod stocks are considered overexploited, and have lost 3% to 49% of their total biomass since 1970 [13]. Thus, this species was listed as ‘vulnerable’ by the International Union for the Conservation of Nature (IUCN), a condition that persists today [14], and is currently protected by strict management. Even so, data from FAO revealed that 1.13 million tons of Atlantic cod and 0.43 million tons of Pacific cod were landed in 2019, indicating that the Atlantic cod (72.3% of the total load recorded for these two species) is by far the most captured [15]. 

Considering the genetic and phenotypic similarities between the Atlantic and Pacific cod species [16,17], it would seem reasonable to transfer part of the fishing pressure from the Atlantic cod towards the Pacific counterpart for a period of time established in accordance with the Pacific cod biomass surveillance data. Anticipating such expectable change in the trade of cod, this study aims to expand the knowledge on the composition of the Atlantic and Pacific cod species edible portion, thereby evaluating the scientific hypothesis that the Atlantic and Pacific cod species present similar composition in their lipid fractions.

## 2. Materials and Methods

### 2.1. Sample Characterization and Preparation 

A total of 45 dried salted cods (2–3 kg of dry weight; *n* = 15 samples/origin) were used in this study. The Atlantic cod was fished in the Atlantic northeast (FAO 27 area) within the Exclusive Economic Zones (EEZ) of Norway (*n* = 15) and Iceland (*n* = 15), while the Pacific cod was caught in the Pacific northeast (FAO 67 area) within the Alaska EEZ (*n* = 15). The dried salted cods used herein were randomly selected among specimens harvested between January and April of 2018, sharing analogous fishing and processing methodologies, and were all collected at Riberalves Company (Carvalhal, Torres Vedras, Portugal).

According to Good Manufacturing Practices of Riberalves, the average time between cod harvesting and the salting and drying processes was two to three months. Salting and drying processes were performed according to commercial procedures for specimens weighing 2–3 kg. 

All samples for the various analyses were taken from the central portion of loins after the five to six days of the drying process had been completed. Skin and bones were manually removed, and the remaining part of muscle tissue (designated hereafter as the edible portion) was cut into thin slices before blending in a food processor (Moulinex, France). Half of the homogenised material was vacuum packed and frozen/stored at −20 °C until further analysis, within one month, while the other half was freeze dried (−60 °C and 2.0 h Pa) up to constant weight, using an Edwards Modulyo freeze dryer (Edwards High Vacuum International, West Sussex, UK), packed and kept at room temperature, and analysed within one month. In all assays, the 45 samples were analysed in duplicate, and the results were accepted when the coefficient of variation between duplicates was below 5%. Whenever duplicates were associated with a higher coefficient of variation, the analytical procedure was repeated. The average value obtained for each sample analysis was used in the statistical analysis (*n* = 45; 15 samples/cod group).

### 2.2. Total Lipids and Fatty Acid Analysis

Total lipids (TL) were extracted according to the method previously established by [18]. Briefly, methanol, chloroform and Milli Q water were added to the sample in a two-step extraction, and after phase separation, lipids were obtained in the chloroform phase. 

Fatty acid methyl esters (FAMEs) were prepared, in duplicate, by acid-catalysed transesterification using the methodology previously described [19]. Briefly, each replicate sample (150 mg of total lipids) was dissolved in 5 mL of acetyl chloride/methanol (1:19 *v*/*v*; Merck Biosciences, Darmstadt, Germany), shaken, and heated (80 °C; 1 h). After cooling, 1 mL of Milli Q water (HPLC-grade) and 2 mL of n-heptane pro analysis (Merck Biosciences, Darmstadt, Germany) were added, and samples were shaken and centrifuged (2300 *g*, 5 min) until phase separation. The organic phase was collected and dried with anhydrous sodium sulphate pro analysis grade (Panreac Química, Barcelona, Spain). Subsequently, an aliquot (2 μL) of the upper phase was injected into a GC.

The resultant methyl esters were applied to a DB-WAX (Agilent Technologies, Santa Clara, CA, USA) capillary column (film thickness, 0.25 μm), 30 m × 0.25 mm i.d., integrated in a Varian Star 3800 CP gas chromatograph (Walnut Creek, CA, USA), equipped with an auto sampler with a split injector (100:1) and a flame ionization detector, both at 250 °C. Helium was used as the carrier gas in the separation of FAMEs. The column temperature was as follows: initial temperature of 180 °C, increased to 200 °C at 4 °C /min, held for 10 min at 200 °C, then increased to 210 °C at 4 °C /min, and maintained at this temperature for 14.5 min. The identification of FAME was achieved by comparing the FAME retention times with those of Sigma–Aldrich standards (PUFA-3, Menhaden oil, and PUFA-1, Marine source from Supelco Analytical). The fatty acid profile is expressed as a percentage of total FAs, while fatty acid partial sums are expressed as mg/g of edible portion). The quantification of each FAME was estimated using the known concentration and signal area of internal standard, using the following formula:

Fatty acid (mg/100 g edible muscle) = 100 × [((mg C21:0) × (FAME area))/(C21:0 area))/(g edible muscle)].

### 2.3. Total Cholesterol (TCHR) and Vitamin E Analysis

The simultaneous determination of TCHR and vitamin E (α-tocopherol) contents was performed as previously described [20]. Briefly, the method involves a direct saponification of the dried salted cod edible portion (500 mg) with a saponification solution (11% *w*/*v* potassium hydroxide in a mixture of ethanol: Milli Q water (55:45 *v*/*v*)), performed at +80 °C for 15 min, followed by a single n-hexane extraction and HPLC analysis of the extract. Quantification was performed by normal-phase HPLC (column Zorbax Rx Sil, 4.6 mm ID × 250 mm, 5 μm particle size, Agilent Technologies Inc., Palo Alto, CA, USA), with UV–VIS photodiode array (cholesterol) and fluorescence (tocopherols) detectors in tandem. The contents of total cholesterol and α-tocopherol were calculate based on the external standard technique from a standard curve of peak vs. compounds concentrations.

### 2.4. Lipid Quality Ratios and Indices

The nutritional quality of lipids was assessed considering the indices of: peroxidability (PI) [21], atherogenicity (AI) and thrombogenicity (TI) [22] and the ratios: hypocholesterolaemic/hypercholesterolaemic (h/H) [23], polyunsaturated/saturated FAs (P/S) [24], and n3/n6; which were calculated according to the equations presented below:

PI = (% monoenoic × 0.025) + (% dienoic × 1) + (% trienoic × 2) + (% tetraenoic × 4) + (% pentaenoic × 6) + (% hexaenoic × 8);

AI = (C12:0 + 4 × C14:0 + C16:0)/[(∑MUFA + ∑(n-6) + ∑(n-3)]

TI = (C14:0 + C16:0 + C18:0)/[(0.5 × (∑MUFA)) + (0.5 × (∑n-6)) + (3 × (∑n-3) + (∑n-3)/(∑n-6)];

P/S = [(18:2 n-6) + (18:3 n-3)/(14:0 + 16:0 + 18:0)];

n-3/n-6 [(∑n-3)/(∑n-6)];

h/H = [(C18:1 *cis*-9 + C18:2 n-6 + C18:3 n-3 + C20:4 n-6 + C20:5 n-3 + C22:5 n-3 + C22:6 n-3)/(C14:0 + C16:0)];

where MUFA means monounsaturated fatty acids, while the n-6 and n-3 stand for all the FAs belonging to the n-6 or n-3 families within polyunsaturated fatty acids (PUFA).

### 2.5. Statistical Analysis

Throughout the results and discussion, the term superiority (expressed as %) was calculated as (maximum value–minimum value)/minimum value.

The statistical analysis was accomplished using the PROC MIXED procedure of the Statistical Analysis System (SAS Inst., Cary, NC, USA; version 9.4), considering the species plus origin as single effect. A total of two orthogonal contrasts were used to evaluate the effect of the species (Atlantic cod vs. Pacific cod; A vs. P) and evaluate the origin effect within Atlantic cod (Norwegian vs. Icelandic cod; N vs. I).

The values presented in Table 1 are the least squares means and standard error of mean (LSM and SEM, respectively), while values presented in Table 2 are expressed as average ± standard deviation. Whenever a significant difference was detected in ANOVA, least squares means were compared for alpha = 0.05, using the LSD test adjusted by the Tukey method.

Canonical discriminant analysis (CDA) was applied to the fatty acid profiles in order to discriminate and predict the cod’s origin. Variable selection for CDA was achieved using: (1) the significant variables defined after ANOVA, considering the species plus origin as a single effect (Proc GLM, SAS Inst., Cary, NC, USA; version 9.4); (2) stepwise discriminant analysis with forward selection and a significance level for entering variables of 0.05 (SAS, Proc STEPDISC) to select the variables with a major discriminant capacity; (3) CDA and cross-validation were conducted using Proc DISCRIM from SAS. 

## 3. Results and Discussion

### 3.1. Composition of the Lipid Fraction

The contents of total lipids (TL), total cholesterol (TCHR), fatty acid partial sums and ratios, lipid nutritional indices, and vitamin E contents in the cod edible portions from the Atlantic (harvested in Norway and Iceland) and the Pacific (harvested in Alaska) are shown in Table 1, while the detailed fatty acid profile (expressed as % of total FAs) is depicted in Table 2

#### 3.1.1. Total Lipids and Total Cholesterol Contents

TL and TCHR contents did not reveal significant differences between the cod species, neither between different origins within the Atlantic area (*p* > 0.05). The TL and TCHR content averaged 1.45 g/100 g and 69.6 mg/100 g of the edible portion, respectively. Despite the lack of statistical significance, the TL mean values achieved by the Icelandic (1.8%) and Norwegian (1.1%) specimens may diversely influence their respective texture and flavour attributes after cooking. 

Assuming an estimated average weight loss of 37% due to drying and salting procedures, the TL contents in fresh cod used for the production of the dried salted cod tested in the study would be within the range of TL contents reported by other authors (0.15–0.70 g/100 g of fresh edible portion) [25,26]. It is important to highlight that cod muscle TL content is quite variable, being mainly influenced by age, sex, reproductive status, harvesting season, type and abundance of available food, water depth and temperature [3,27]. Concerning TCHR, the values presented herein for dried salted cod suggest that the corresponding fresh cod edible portion would present a content in the mid-range of values previously reported (39–60 mg/100 g of fresh edible portion) [28,29]. The absence of significant differences on TCHR between different species and different harvesting locations suggests that the cod specimens used in this study were harvested from fish stocks living in similar temperatures, since cholesterol content is positively correlated with annual mean water temperature [30]. Dried salted cod is a poor source of cholesterol, considering the recommendations for daily cholesterol ingestion, which should not exceed 300 mg [31]. To exceed the maximum daily intake, it would require the ingestion of more than 420 g of dried salted cod or 525 g of soaked cod, which is quite improbable on a daily basis.

#### 3.1.2. Fatty Acid Profile

Fatty acids (FAs) are prime structural units of both polar and neutral lipids, as phospholipids and triacylglycerols, which are responsible for 55–56 and 11–12% of cod’s edible portion total lipids, correspondingly [26,32]. The fatty acid profile presented herein includes all FAs present in different lipid classes within the edible portion, however, different lipid classes in a single tissue possess distinct fatty acid profiles [32]. Despite being a minor lipid class among the fish edible portion, triacylglycerols are probably the main lipid class displaying changes in their fatty acid profile, while the FAs present in polar lipids are quite stable [32].

Among individual FAs, C22:6 n-3 (docosahexaenoic acid or DHA) and C20:5 n-3 (eicosapentaenoic acid or EPA) were the most abundant within polyunsaturated fatty acids (PUFA), accounting for 28.2–33.4 and 15.1–20.1% of total FAs, respectively. C16:0 and C18:0 (palmitic and stearic acids, respectively) were responsible for 20.5–24.0 and 4.7–5.3% of total FAs and were the most abundant fatty acid among the saturated fatty acids (SFA). The most concentrated monounsaturated fatty acids (MUFA) were oleic (C18:1 *cis*-9) and *cis*-vaccenic (C18:1 *cis*-11) acids, which were accountable for 7.0–9.3 and 3.3–3.6% of the total FAs, respectively. Together, the two predominant FAs from SFA, MUFA and PUFA were responsible for 85.3–87.2% of total FAs in the cod’s edible portion, independently of the species and origin. The main FAs and their contribution to the total content in dried salted cod, in both Atlantic and Pacific cod species, are in full agreement with data previously published for fillets of both species (78.3–87.5% of total FAs) [26,29,30,32,33]. Regarding EPA and DHA (long chain n-3 PUFA), they are simultaneously the FAs with the greatest biological value concerning human health and the ones more prone to oxidation, due to their unsaturation degree [21]. The amount of EPA plus DHA in dried salted cod (43.3–49.6% of total FAs) is similar to the values previously presented for fresh cod (44–51.7% of total FAs) [26,29,30,32,33]. Such similarity in the proportion of EPA plus DHA in both dried salted and fresh cod edible portions suggests that: (1) the drying and salting processes do not affect the fatty acid profile; or (2) the endogenous antioxidant systems in cod’s muscle tissue are efficient in the inhibition of lipid oxidation.

The differences between Atlantic and Pacific cod species and within Atlantic cod harvesting locations (Norway and Iceland) occurred in 9 and 10 FAs (*p* < 0.05), respectively. The Norwegian fish displayed higher contents of C20:4 n-6, C20:5 n-3 and C22:5 n-3, but lower contents of C15:0, C16:0, C16:1 *cis*-9, C17:1 *cis*-9, C18:1 *cis*-9, C20:1 *cis*-11 and C18:4 n-3 than the Icelandic counterparts. Such results suggest differences in the proportion of polar and neutral lipid fractions, which is sustained by the FAs differential distribution between such fractions, within the edible portion., since C16:0, C16:1 *cis*-9, C17:1 *cis*-9, C18:1 *cis*-9, C20:1 *cis*-11 and C18:4 n-3 are preferentially stored in triacylglycerols, whereas C20:4 n-6 and C20:5 n-3 are essentially used as structural elements of phospholipids [29,32]. The comparison of cod species shows that the Atlantic cod edible portion presents higher contents of C14:0, C15:0, C16:1 *cis*-7, C20:1 *cis*-13, C16:2 n-4 and C20:5 n-3, and lower contents of C18:0, C17:1 *cis*-9 and C22:6 n-3 in comparison with the Pacific counterpart. These differences do not seem to be dependent on different proportions of polar and neutral lipid fractions. Therefore, such differences could be dependent on different FA bioavailability between different feed webs.

The magnitude of differences observed between harvesting locations in the Atlantic cod (ten significant differences among individual FAs, accountable for 61.6% of total FAs), was unexpectedly at a similar level to that observed between the cod species (nine significant differences among individual FAs, accountable for 63.2% of total FAs). Such similarities suggest that the muscle fatty acid composition of gadidae species is quite similar. This fact indicates genetic likenesses between the Atlantic and Pacific species, which is in agreement with the assumption that the Pacific cod is the result of speciation that occurred by the migration of the Atlantic cod into the Artic and Pacific oceans after the opening of the Bering Strait in the mid-Pliocene (3.0 to 3.5 million years ago) [17].

Despite the observed differences in individual FAs between cod species, no distinction (*p* > 0.05) was found on FA partial sums (i.e., SFA, MUFA and PUFA and n-3 and n-6 families), nor in the FA ratios (P/S and n3/n6). Once again, the similarities observed between cod species are noteworthy, showing that they share equal proportions of prime FAs groups and families. However, the comparison of Norwegian and Icelandic cod evidenced that the latter displayed significant higher total MUFA contents (*p* = 0.006) and tended to present higher total SFA contents (*p* = 0.050), which is in agreement with the TL contents. Even so, no significant differences were observed between fatty acid ratios (P/S and n3/n6).

Concerning the lipid quality indices and the h/H ratio, Norwegian cod presented simultaneously healthier h/H ratio (2.81 vs. 2.19), and AI index (0.39 vs. 0.47), yet displayed worse PI (3.79 vs. 3.31) than the Icelandic counterpart. On the other hand, Pacific cod presented the healthier h/H ratio and AI, but worse PI, and no significant differences on TI.

Fish is the major source of both EPA and DHA in the human diet, consequently, EPA plus DHA are of utmost importance when evaluating its FAs nutritional quality [34,35,36,37]. The highest and lowest EPA plus DHA proportions were observed among Atlantic cod, with the Icelandic and Norwegian cod reaching the highest and lowest values (49.6 vs. 43.4% of total FA), while the Pacific cod attained a halfway value of EPA plus DHA (48.5% of total FAs). However, a portion of 100 g of dried salted cod (or 125 g of soaked cod), independently of the origin or species, slightly exceeds the recommendations established by the European Food Safety Authority [38], which established the daily intake of EPA plus DHA of 250 mg, based on cardiovascular considerations. The highest EPA plus DHA content was obtained in the Pacific cod (302.3 mg/100 g of edible portion), while the Icelandic and Norwegian cods presented 284.4 and 283.8 g/100 g of edible portion, correspondingly.

#### 3.1.3. Vitamin E Content

Concerning vitamin E, α-tocopherol was the only tocochromanol detected on the cod edible portion. Its content was not influenced (*p* > 0.05) by origin within Atlantic area, but it was significantly affected (*p* < 0.05) by species, with higher level in the Pacific than in the Atlantic cod (8.04 vs. 4.94 µg/g). α-Tocopherol is exclusively synthesized by plants and other photosynthetic organisms [39], therefore, differences in the edible portion should be a consequence of differences in dietary availability of α-tocopherol. The higher α-tocopherol content observed in the Pacific cod relative to the Atlantic cod will provide higher antioxidant protection, counterbalancing the Pacific cod fatty acid profile increased susceptibility to peroxidation, estimated by the PI (3.74 vs. 3.55). The antioxidant protection provided by α-tocopherol (a superiority of 62.7%) is regarded as advantageous from the nutritional point of view because fewer nutritional losses are expected as a consequence of oxidation [40,41]. However, such expected lower susceptibility to lipid oxidation, may also decrease the generation of volatile derivatives, formed by lipid oxidation and implicated in the flavour after cooking. Aldehydes (such as pentanal and hexanal) and alcohols (such as 1-penten-3-ol or *cis*-2-penten-1-ol) obtained from C18:2 n-6 and C18:3 n-3 oxidation, respectively, have been regarded as positive sensorial discriminant factors [42]. Despite its possible negative influence in dried salted cod sensorial attributes, the higher α-tocopherol content observed in Pacific cod has to be regarded as a nutritional positive attribute, since it is the prime lipid-soluble antioxidant in human tissues [43].

### 3.2. Discriminatory Ability of Intramuscular Fatty Acid Pattern

**Figure 1:**
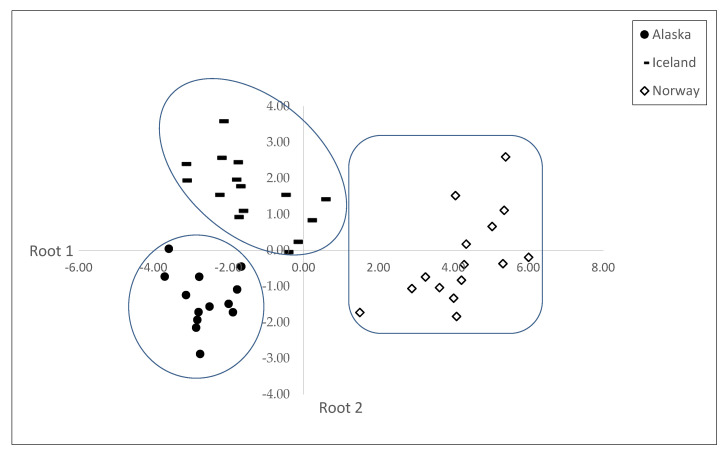
Plot of the discriminant functions (root 1 vs. root 2) for classification of cod varieties (Alaska, Iceland and Norway).

Canonical discriminant analysis was applied to the fatty acid profiles in order to discriminate the cod’s origin. The results of canonical discriminant analysis, loadings of the correlation matrix and discriminant functions are depicted in Table 3. A stepwise forward discriminant analysis was previously applied in order to select the most relevant variables for classification. In this procedure, variables that contribute with the most discriminatory power were selected. The application of canonical discriminant analysis to selected variables produced two canonical discriminant functions, which maximized the ratio between class variance whilst minimising the ratio within class variance. The coefficients obtained for each variable are presented in Table 3. A larger coefficient corresponds to a greater contribution of the respective variable to the discrimination between groups. For origin differentiation, the first two canonical discriminant functions were selected (Figure 1). The recognition ability of the discriminant model was evaluated by the correct classifications of 100% during the modelling step, allowing the differentiation of the three cod varieties. Afterwards, the prediction ability was carried out with a cross-validation method, in which one sample at a time was removed from the training set and considered as a test set. A correct classification of 100% was obtained for all cod varieties (100% accuracy), confirming the high sensitivity and specificity of the class model (Table 4). Considering the data presented in Figure 1, presenting the discriminant roots 1 and 2, the Pacific cod caught in Alaska was located in the left inferior quadrant of the plot (embracing the negative values of both roots). The Norwegian cod was located in the right half of the plot (comprising root 1 positive values), but including the upper and lower quadrants (root 2 positive and negative values). The Icelandic cod was predominantly distributed in the upper left quadrant (root 1 negative values and root 2 positive values), but two fish of the Icelandic cod were positioned in the upper right quadrant (roots 1 and 2 positive values). Therefore, cod samples from different origins were effectively discriminated by their fatty acid profile. The cod’s flesh FAs with the highest discriminant power were the C14:0, C16:0, C18:1 *cis*-9, C20:5 n-3 and C22:5 n-3 in root 1 and the C22:6 n-3, C14:0, C18:1 *cis*-9, C22:5 n-3 and C18:0 in root 2.

## 4. Conclusions

The cod species and the harvesting locations within Atlantic cod had no significant influence on the total lipid and total cholesterol contents of the cod’s edible muscle (flesh). Differences observed between the cod species on individual fatty acids occurred at a similar extent to those observed between harvesting locations for the Atlantic cod. Pacific cod presented higher contents of EPA plus DHA and α-tocopherol than the Atlantic cod. The fatty acid profile was reliable to discriminate different cod species and even different harvesting locations within the Atlantic cod.

When considering the composition of the lipid fraction, the replacement of Atlantic cod by Pacific cod in the production of salted dried cod does not seem to affect the nutritional value.

## Figures and Tables

**Table 1:** Total lipid (TL), total cholesterol (TCHR) and α-tocopherol contents, fatty acid partial sums, fatty acid ratios and lipid quality indices of dried salted cod edible portion obtained from the Atlantic and Pacific cod species and different harvesting locations within the Atlantic cod.

	Atlantic Cod		Pacific Cod		Statistics	
	Norway	Iceland	Alaska	SEM	A vs. P	N vs. I
TL (g/100 g)	1.1	1.8	1.3	0.559	0.889	0.498
TCHR (mg/100 g)	70.44	68.45	70.02	2.996	0.875	0.843
α-tocopherol (µg/g)	4.90	4.97	8.04	0.677	0.001	0.996
Partial sums (mg/g of edible portion)						
SFA	1.573	2.076	1.806	0.176	0.934	0.050
MUFA	0.885	1.212	1.023	0.080	0.796	0.006
PUFA	3.263	3.266	3.405	0.308	0.712	0.996
n6	0.201	0.215	0.218	0.021	0.686	0.654
n3	3.025	3.010	3.151	0.286	0.705	0.971
Fatty acid ratios and lipid quality indices						
P/S ^1^	0.047	0.043	0.051	0.003	0.113	0.353
n3/n6 ^2^	15.09	14.67	14.84	1.029	0.819	0.863
h/H ^3^	2.812	2.196	2.666	0.065	0.049	<0.001
AI ^4^	0.387	0.465	0.383	0.011	0.002	<0.001
TI ^5^	0.015	0.019	0.017	0.001	0.989	0.003
PI ^6^	3.791	3.313	3.745	0.049	0.003	<0.001

SEM—standard error of the mean; A vs. P—Atlantic cod vs. Pacific cod; N vs. I—Norwegian vs. Icelandic cod. ^1^ P/S = [(18:2 n-6 + 18:3 n-3)/(14:0 + 16:0 + 18:0)]; ^2^ n3/n6= [(∑n-3)/(∑n-6)]; ^3^ h/H hypocholesterolaemic/hypercholesterolaemic ratio; ^4^AI atherogenicity index; ^5^ TI thrombogenicity index; ^6^ PI peroxidability index.

**Table 2:** Fatty acid profiles of dried salted cod edible portion obtained from the Atlantic and Pacific cod species and from different harvesting locations within the Atlantic cod (expressed as g/100 g of total fatty acids; mean ± standard deviation).

Fatty Acids	Atlantic Cod		Pacific Cod	Contrasts	
	Norway	Iceland	Alaska	A vs. P	N vs. I
C14:0	1.82 ± 0.34	1.87 ± 0.44	1.23 ± 0.20	<0.001	0.686
C15:0	0.31 ± 0.03	0.34 ± 0.04	0.28 ± 0.03	<0.001	0.024
anteiso-C16:0	0.14 ± 0.08	0.18 ± 0.11	0.13 ± 0.01	0.398	0.325
C16:0	20.5 ± 1.44	24.0 ± 0.86	21.9 ± 0.22	0.487	<0.001
C17:0	0.21 ± 0.15	0.23 ± 0.15	0.20 ± 0.01	0.579	0.586
C18:0	4.72 ± 0.45	5.07 ± 0.62	5.337 ± 0.04	0.006	0.059
C16:1 *cis*-9	1.24 ± 0.75	1.86 ± 0.62	1.36 ± 0.18	0.069	<0.001
C16:1 *cis*-7	0.68 ± 0.50	0.63 ± 0.38	0.40 ± 0.03	0.017	0.721
C17:1 *cis*-9	0.06 ± 0.03	0.09 ± 0.05	0.13 ± 0.08	0.041	0.004
C18:1 *cis*-13	0.40 ± 0.04	0.51 ± 0.43	0.36 ± 0.23	0.261	0.258
C18:1 *cis*-11	3.44 ± 0.40	3.63 ± 0.55	3.26 ± 0.51	0.066	0.276
C18:1 *cis*-9	7.03 ± 0.59	9.26 ± 0.88	8.26 ± 0.69	0.588	<0.001
C20:1 *cis*-13	1.35 ± 0.52	1.95 ± 1.26	0.15 ± 0.08	0.012	0.200
C20:1 *cis*-11	1.69 ± 0.33	2.43 ± 1.12	2.34 ± 0.24	0.212	0.006
C22:1 *cis*-11	0.35 ± 0.21	0.53 ± 0.38	0.45 ± 0.22	0.952	0.096
C16:2 n-4	0.65 ± 0.08	0.62 ± 0.08	0.55 ± 0.05	<0.001	0.329
C18:2 n-6	1.02 ± 0.12	1.20 ± 0.47	1.26 ± 0.35	0.111	0.087
C20:4 n-6	2.44 ± 0.35	2.07 ± 0.43	2.20 ± 0.64	0.729	0.038
C16:4 n-3	0.15 ± 0.11	0.23 ± 0.13	0.17 ± 0.05	0.489	0.069
C18:3 n-3	0.25 ± 0.04	0.26 ± 0.14	0.23 ± 0.11	0.451	0.749
C18:4 n-3	0.54 ± 0.09	0.63 ± 0.08	0.53 ± 0.22	0.150	0.045
C20:4 n-3	0.42 ± 0.22	0.37 ± 0.24	0.35 ± 0.19	0.293	0.300
C20:5 n-3 (EPA)	20.1 ± 1.91	15.2 ± 1.57	15.1 ± 1.54	<0.001	<0.001
C22:5 n-3	2.03 ± 0.88	0.79 ± 0.46	1.22 ± 0.63	0.291	<0.001
C22:6 n-3 (DHA)	29.5 ± 2.89	28.2 ± 2.34	33.4 ± 3.26	<0.001	0.177

A vs. P—Atlantic cod vs. Pacific cod; N vs. I—Norwegian vs. Icelandic cod.

**Table 3:** Results of the canonical discriminant analysis: loadings of the correlation matrix between predictor variables (standardized canonical coefficients) and discriminant functions (roots 1, 2 and 3), and some statistics for each function.

Variables	Root 1	Root 2
C14:0	−1.0922	0.8489
C15:0	0.3042	−0.1335
C16:0	0.8185	0.5272
C18	−0.2992	−0.3687
C16:1 *cis*-9	0.2795	−0.1934
C18:1 *cis*-9	0.8031	0.6734
C20:1 *cis*-11	0.1487	0.4687
C16:2 n-4	−0.5911	−0.3427
C18:4 n-3	0.4235	−0.3404
C20:5 n-3 (EPA)	−0.8695	−0.0030
C22:5 n-3 (DPA)	−0.9837	0.6297
C22:6 n-3 (DHA)	0.3468	−1.2826
Statistics		
Canonical R	0.9271	0.7626
Eigenvalue	6.1167	1.6384
Cumulative proportion	0.7887	1.0000
Probability	<0.001	<0.001

**Table 4:** Classification matrix of cross-validation for cod origin (Number of observations and percent classified into variety).

Variety	Number of Observations and Percentage Classified into Variety		
	Iceland	Norway	Alaska
Classified as Iceland	15	0	0
Classified as Norway	0	15	0
Classified as Alaska	0	0	15
Total	15	15	15
Correct classification (%)	100%	100%	100%

## Data Availability

Data are available on request to the corresponding author.
